# Exosomes derived from human placental mesenchymal stem cells enhanced the recovery of spinal cord injury by activating endogenous neurogenesis

**DOI:** 10.1186/s13287-021-02248-2

**Published:** 2021-03-12

**Authors:** Wenshu Zhou, Marta Silva, Chun Feng, Shumei Zhao, Linlin Liu, Shuai Li, Jingmei Zhong, Wenhua Zheng

**Affiliations:** 1 Centre of Reproduction, Development and Aging, Institute of Translational Medicine, Faculty of Health Sciences, University of Macau, Room 4021, Building E12, Taipa, Macau, SAR China; 2 Yunnan Key Laboratory of Primate Biomedical Research, Institute of Primate Translational Medicine, Kunming University of Science and Technology, Kunming, 650500 Yunnan China; 3 First People’s Hospital of Yunnan Province, Psychiatry Department, Kunming, 650032 Yunnan China

**Keywords:** Spinal cord injury, Mesenchymal stem cell-derived exosomes, Motor function, Autonomic function, Neurogenesis

## Abstract

**Background:**

Spinal cord injury (SCI) is a debilitating medical condition that can result in the irreversible loss of sensorimotor function. Current therapies fail to provide an effective recovery being crucial to develop more effective approaches. Mesenchymal stem cell (MSC) exosomes have been shown to be able to facilitate axonal growth and act as mediators to regulate neurogenesis and neuroprotection, holding great therapeutic potential in SCI conditions. This study aimed to assess the potential of human placental MSC (hpMSC)-derived exosomes on the functional recovery and reactivation of endogenous neurogenesis in an experimental animal model of SCI and to explore the possible mechanisms involved.

**Methods:**

The hpMSC-derived exosomes were extracted and transplanted in an experimental animal model of SCI with complete transection of the thoracic segment. Functional recovery, the expression of neural stem/progenitor cell markers and the occurrence of neurogenesis, was assessed 60 days after the treatment. In vitro, neural stem cells (NSCs) were incubated with the isolated exosomes for 24 h, and the phosphorylation levels of mitogen-activated protein kinase kinase (MEK), extracellular signal-regulated kinases (ERK), and cAMP response element binding (CREB) proteins were assessed by western blot.

**Results:**

Exosomes were successfully isolated and purified from hpMSCs. Intravenous injections of these purified exosomes significantly improved the locomotor activity and bladder dysfunction of SCI animals. Further study of the exosomes’ therapeutic action revealed that hpMSC-derived exosomes promoted the activation of proliferating endogenous neural stem/progenitor cells as denoted by the significant increase of spinal SOX2^+^GFAP^+^, PAX6^+^Nestin^+^, and SOX1^+^KI67^+^ cells. Moreover, animals treated with exosomes exhibited a significative higher neurogenesis, as indicated by the higher percentage of DCX^+^MAP 2^+^ neurons. In vitro, hpMSC-derived exosomes promoted the proliferation of NSCs and the increase of the phosphorylated levels of MEK, ERK, and CREB.

**Conclusions:**

This study provides evidence that the use of hpMSC-derived exosomes may constitute a promising therapeutic strategy for the treatment of SCI.

## Background

Spinal cord injury (SCI) is a serious debilitating central nervous system (CNS) injury that can lead to temporary or permanent damage of motor, sensory and autonomic functions [1, 2]. With a worldwide incidence gradually increasing, SCI has become a major burden on the society, patients, and caregivers [3], because despite the current understanding of its complex pathophysiology, available treatments lack effectiveness and can only improve symptoms and reduce complications [4]. SCI induces axonal damage, demyelination, and neuronal loss while limiting the activation of endogenous neurogenesis [5]. Thus, the development of strategies aiming to promote neuronal regeneration via reactivation and recruitment of endogenous neural progenitor cells (NPCs) may hold great potential [6]. In the last years, much effort has been put towards the development of innovative cell replacement strategies using mesenchymal stem cells (MSCs), in order to promote the recovery of patients with SCI [7]. MSCs are a multipotent population with self-renew capacity and the potential to differentiate into different cell types, with MSCs derived from the placenta being reported to have a higher proliferation ability compared with bone marrow MSCs [8, 9]. In spite of their therapeutic potential, there are some obvious disadvantages associated to the use of MSCs in the treatment of SCI. Specifically, the survival rate of the transplanted stem cells is very low due to severe inflammatory responses or other factors in the injured microenvironment [10–12]. Additionally, the scar-forming gliosis prevents the integration, differentiation, and axon outgrowth of grafted stem cells in the lesion area [13, 14]. Moreover, there are also still some concerns regarding the safety of these cells [15, 16]. More recently, accumulating evidence suggests that the therapeutic action of MSCs is due to paracrine mechanisms that mainly occur via secretion of exosomes [17–19]. Exosomes are nanosized vesicles with a phospholipid bilayer involved in cell-to-cell communication, cell signaling, and being able to alter cell or tissue metabolism at short or long distances in the body [20, 21]. MSC exosomes enclose lipids (lipids or lipid rafts, cholesterol, sphingolipid ceramide, lipid raft marker protein-1, phosphoglycerides) and nucleic acids (such as miRNA, coding RNA, non-coding RNA, DNA) [22–24]. At the same time, they have common marker proteins on their surfaces, such as membrane proteins (CD9, CD63, CD81, CD82), proteins involved in the sorting and transport of intercellular complexes (Alix, Tsg101), membrane transport fusion eggs (GTPases, annexins, flotillin, Rab protein), and heat shock protein (HSP70, HSP90) [25, 26]. Compared with MSCs, exosomes are safer, more stable, and have lower immunogenicity [17]. Moreover, they possess a longer half-life in circulation and are able to cross the brain-blood barrier, enabling their use as delivery carriers in the treatment of CNS diseases [27–29]. Different studies of MSC exosomes for spinal cord injury repair report their ability to facilitate axonal growth, induce angiogenesis, regulate inflammatory and immune responses, inhibit apoptosis, and maintain the integrity of the blood-spinal cord barrier (BSCB) [17, 30, 31]. Based on these evidence, this study aimed to assess the effect of exosomes secreted by human placental MSCs (hpMSCs) on the functional recovery and reactivation of endogenous neurogenesis in an experimental animal model of SCI with a complete transection of the thoracic segment and to explore the possible mechanisms involved.

## Materials and methods

### Culture of hpMSCs

Human placenta samples were collected during cesarean section procedures under aseptic surgical conditions. The use of human placenta samples was approved by the ethics committee of Kunming University of Science and Technology, and the written informed consent was obtained before clinical sampling. Placenta-derived mesenchymal stem cells were isolated as previously described [32]. Briefly, placenta samples were repeatedly rinsed with phosphate buffered saline (PBS) containing penicillin and streptomycin, until they were free of blood. Then the samples were cut into 0.5~1 cm pieces and transferred (approximately 10 g) to a tube with 10 ml working digestion solution with 100 U/ml collagenase type I, 1.5 μg/ml DNase I, and 2.4 U/ml dispase in serum-free Dulbecco’s modified Eagle’s medium (DMEM). After digestion for 45 min, 30 ml of serum-containing medium (SCM), composed of DMEM low glucose (Gibco, 10567014) with 10% fetal bovine serum (FBS, MSC010, Excell) and 1% NEAA were added. The digested samples were then filtered with 40 μm cell strainers (Corning Falcon™) and centrifuged for 5 min at 340*g*. The enriched cells were resuspended by SCM and subsequently incubated in 37 °C, 5% CO_2_ humidified atmosphere. Two days after the initial plating, non-adherent cells were removed by washing with PBS, and the fresh medium was changed every 3 days. Once colonies of fibroblast-like cells appeared and had up to 90% confluency, the cells were detached using 0.25% trypsin-EDTA (Gibco, 25200056) and expanded in serum-containing medium (SCM) and serum-free medium (SFM) (10% KOSR (Gibco, N10828-028), 1% NEAA (Gibco, 231-791-2), 10 ng/ml FGF (Millipore, GF003AF), and 20 ng/ml EGF (Millipore, 01-107)).

### Tri-lineage differentiation of hpMSCs

Tri-lineage differentiation of hpMSCs into adipocytes, osteocytes, and chondrocytes was carried out using previously described techniques [8]. Adipogenic differentiation was initiated in confluent cultures of MSCs using Stempro adipogenesis differentiation kit (Gibco, A1007001). After 21 days, adipogenic differentiation was detected by staining the lipid droplets with Oil Red O (Solarbio, G1262). Osteogenic differentiation was induced in confluent cultures of MSCs using stempro osteogenesis differentiation kit (Gibco, A1007201). After 21 days, mineralization was detected by staining with Alizarin Red S (Solarbio, G1450). Chondrogenic differentiation was achieved using the stempro chondrogenesis differentiation kit (Gibco, A1007101). Similarly, chondroitin was assessed by staining Alcian Blue (Solarbio, G2541) after differentiation 21 days.

### Flow cytometry analysis of hpMSCs

To test the multipotency of MSCs, cells at passage 3, cultured both in SCM and SFM, were analyzed by flow cytometry. The samples were first incubated with the following antibodies: CD44 PE-A, CD73 APC-A, CD90 FITC-A, CD105 per-CP-Cy, CD11b PE-A, CD19 PE-A, CD45 PE-A, and HLA-DR PE-A (DB, Cat. no. 562245). After 30 min of incubation at room temperature, cells were analyzed by flow cytometry using BD FACSAria™II.

### Isolation of exosomes secreted by hpMSCs

Exosomes were recovered from hpMSCs medium using both polyethylene glycol (PEG) and ultracentrifugation-based approaches as previously described [22, 33]. In the PEG-based approach, cell culture medium was collected and centrifuged at 300 g for 30 min at 4 °C to remove any cell debris. Fifty percent PEG solution was prepared using deionized water and subsequently centrifuged at 3000 g for 30 min at 4 °C. The supernatant was then filtered through a 0.22-μm syringe filter and mixed with the MSC medium at 1:9 ratio. After incubation at 4 °C for 30 min, the samples were centrifuged at 3000 g for 10 min and exosome pellets were collected and stored in − 80 °C freezer for further analysis. In the ultracentrifugation approach, hpMSCs were cultured for 48 h in SFM and the cell supernatants were collected, and consecutively centrifuged at 300*g* for 10 min and 10,000*g* for 20 min at 4 °C to remove cell debris and apoptotic bodies. After that, the cell supernatants were ultra-centrifuged at 100,000*g* for 70 min at 4 °C and the precipitate was rinsed with PBS. Finally, high purity exosomes were obtained by a second round of ultracentrifugation at 100,000*g* for 70 min at 4 °C, reconstituted in PBS, and stored in − 80 °C freezer for further analysis.

### Transmission electron microscopy (TEM)

Transmission electron microscopy (TEM) was used to assess the morphology of the isolated exosomes [34, 35]. Briefly, exosome suspensions (10 μl) were applied to copper mesh grids, allowed to adsorb to the grid for 1 min, and a filter paper was used to remove the excess exosomes. For negative staining, 1% of Uranyl Acetate (10 μl) was added dropwise to the grid for 1 min and allowed to dry for 10 min at room temperature. TEM examination was performed using high-resolution transmission electron microscope (HRTEM) at 80 kv–120 kv. This experiment was performed at the Institute of Medical Biology, Chinese Academy of Medical Sciences, Kunming.

### Western blot

Western blot was performed to assess the presence of the typical exosome markers CD63 and TGS101 and the phosphorylation levels of mitogen-activated protein kinase kinase (MEK), extracellular signal-regulated kinases (ERK), and cAMP response element binding (CREB) proteins [36]. Cells and exosomes were successively lysed with RIPA buffer and 5 × SDS loading buffer. The protein concentration was determined using the BCA assay kit (Beyotime, P0010). After electrophoresis, proteins were transferred to 0.22 μm PVDF membranes for 1 h. The PVDF membranes were then blocked for 2 h at room temperature with blocking buffer (5% skimmed milk in TBST), and incubated overnight with the primary antibodies (1:1000) at 4 °C. Primary antibodies included CD63 (Abcam, ab134045), Tgs101 (Abcam, ab125011), Phospho-MEK1/2 (CST, 9154) P-ERK (CST, 9101), P-CREB (CST, 9198), and GAPDH (SAB, 21612). After being washed with TBST for three times (10 min each time), the membranes were incubated for 1 h with the secondary antibody (1:5000) at room temperature. Blots were detected using enhanced chemiluminescence.

### Spinal cord injury experimental model

Female Sprague-Dawley (SD) rats, aged 7–8 weeks and weighting 200–220 g at the time of surgery, were used to perform spinal cord injury. All animals were maintained under specific pathogen-free (SPF) conditions and housed under controlled temperature (24–26 °C), humidity, and lighting (12 h light/dark cycle) in the animal facility of Kunming University of Science and Technology. Food and water were available ad libitum throughout the experiment. The experimental procedures were approved by the Lab Animal Care and Use Committee of Kunming University of Science and Technology. The rats were anesthetized with 4% chloral hydrate (10 ml/kg) and placed in the prone position on the operating table. Using aseptic technique, the spinal cord was exposed at the T11 vertebral level via laminectomy as previously described [37]. This step was followed by the complete transection of the spinal cord. The twitching of both hind limbs and paralysis of the animals were indicative of the successful induction of the experimental model.

### Injection of hpMSC-derived exosomes

Animals were randomly divided into two groups: control and exosomes groups (*n* = 6 per group). hpMSC-derived exosomes were transplanted by tail vein injection at 0 (1 h after the operation) and 2 weeks (50 μg total protein of exosome precipitate in 100 μl PBS/rat), taking into consideration the dose used in previous studies [38–40]. Animals from the control group received equal amounts of intravenous PBS. After the injection, penicillin (20,000 U) was administered intraperitonially to prevent infection, and urine was drained by massaging the bladder 2 times a day.

### Behavioral assessment

Behavioral evaluation of the hindlimb locomotor activity was performed using the Beattie-Beattie-Bresnahan (BBB) scores method before the surgery, 1 day and every week after the operation until the end of the experiment [41, 42]. At day 60, after exosomes or PBS injection, the animals were individually placed in 30 × 16 cm cages with filter paper sheets covering the whole floor area in order to measure the voiding pattern [43]. After 1 h, the filter papers were collected, and each urine spot was scanned by MetaMorph image analysis software (Molecular Devices) and calibrated. The diameter of each urine spot was measured using the “trace region” tool. Overlapping urine spots were counted as one, unless the spot had a visible and distinct circumference that could be distinguished from the other spot(s) [44]. The area of the urine spots was quantified.

### Tissue samples preparation

At the end of the experiment, the animals were sacrificed by transcardial perfusion of 4% paraformaldehyde (4% PFA) and the spinal cords were dissected and fixed for 48 h at 4 °C. After fixation, the samples were dehydrated and embedded in OCT. Coronal 0.5 mm sections were cut and kept at − 20 °C until further analysis.

### Neural stem cells culture and treatment

Neural stem cells (NSCs) were obtained and cultured as previously described [45]. Briefly, NSCs were cultured in plates coated with gelatin (0.05%) and laminin (5 μg/ml) and NSC culture medium containing neurobasal media, including 2xB27, 1xN2, and 1% NEAA (Sigma), 1% Glutamax (Sigma), 3 mM CHIR99021, 5 mM SB431542, 10 ng/ml bFGF, and 1000 U/ml hLIF (Millipore). Trypsin 0.025% was used to digest NSCs when passaging to 1:4 every 3 to 4 days. NSCs were seeded in 8-well plates with NSC culture medium for 48 h. The cells were then incubated with exosomes at a concentration of 10 μg/ml of exosomes for 24 h, as previously reported [46].

### Exosomes uptake by NSCs

Exosomes uptake by NSCs was assessed by labeling the exosomes with a green fluorescent dye (PHK67, Sigma) according to the instructions provided by the manufacturer. Labeled exosomes were then incubated with NSCs at 37 °C for 24 h. After this period, the cells were washed with PBS, fixed with 4% PFA, and the nuclei were stained with 4′,6-diamidino-2-phenylindole (DAPI) (Sigma-Aldrich, 32670). Exosomes were observed by confocal microscopy (Carl Zeiss LSM880 Confocal with Airyscan module).

### Immunofluorescence analysis

For immunofluorescence, cells or tissue sections were incubated with 0.2% Triton X-100 (Gibco) for 30 min and then were washed with PBS and incubated with blocking buffer composed of 3% BSA (Sigma-Aldrich, A9647) in PBS for 30 min at room temperature. The samples were then incubated with primary antibody overnight at 4 °C (SOX2 (Millipore, MAB5603, 1:400), PAX6 (RD, MAB1260, 1:1000); SOX1 (R&D, AF3369, 1: 400), NESTIN (Millipore, MAB5922, 1:400); MAP-2 (Millipore, MAB5622,1:600); GFAP (Sigma, G9269, 1:2000), DCX (Millipore, MABN707, 1:500), TUJ1 (Covance, MRB435P, 1:1000), NeuN (Millipore, ABN78,1:500), P-CREB (CST, 9198)). The following day, the samples were washed with PBS and incubated with the corresponding secondary antibody (Alexa Fluor 488 anti-mouse or 594 anti-rabbit (Invitrogen,1: 500)) for one hour at room temperature. Nuclei were counterstained with DAPI (Sigma-Aldrich, 32,670) for 15 min at room temperature. The cells were photographed using a confocal microscope (Carl Zeiss LSM710 Confocal). Tissue sections were photographed using a microscope (Leica, SP8) and the number of cells was counted using Image J software. The percentage of positive cells was calculated from the total nucleus population. All studies were performed for a minimum of 3 sections per sample, with 6 animals in each group.

### Statistical analysis

Data analysis was performed using GraphPad Prism 5 software. Each experiment was repeated three times, and all data are expressed as the mean ± SD. The statistical significance of multiple groups was evaluated using unpaired *t* test. A *p* < 0.05 was considered statistically significant.

## Results

### hpMSCs were successfully isolated from the placenta

**Fig. 1:**
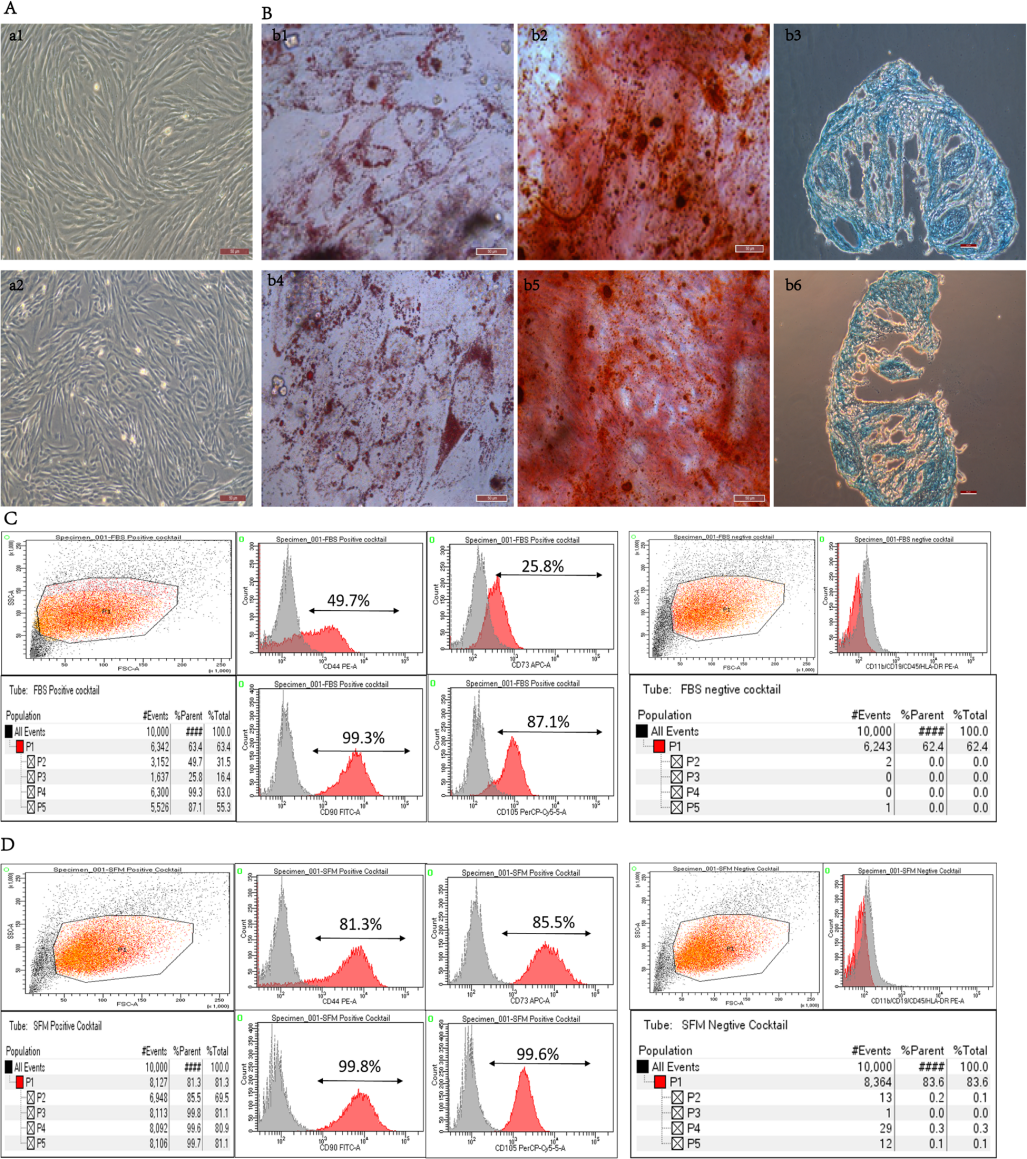
hp-MSCs were successfully isolated from the placentas. **A** Morphological characteristics of MSCs isolated from human placental tissue (scale bars 50 μm) in SCM (a1) and SFM (a2). **B** Three-lineage differentiation of hpMSCs (scale bars 50 μm). Representative images of SCM-cultured MSCs before and after adipogenic (Oil Red O staining) (b1), osteogenic (von Kossa staining) (b2) and chondrogenic (Alcian blue staining) (b3) differentiation; Representative images of SFM-cultured MSCs before and after adipogenic (Oil Red O staining) (b4), osteogenic (von Kossa staining) (b5) and chondrogenic (Alcian blue staining) (b6) differentiation. **C** Surface markers expression of hpMSCs cultured in SCM. D Representative pictures of flow cytometric analysis of the surface marker expression in hpMSCs cultured in SFM. Each experiment was performed in triplicate

Mesenchymal stem cells from full-term fetal placental tissue were successfully isolated. Adherent cells maintained in both culture mediums (SCM and SFM) exhibited a relatively homogeneous and fibroblast-like morphology as previously described (Fig. 1A) [47]. Evaluation of the differentiation potential of isolated hpMSCs into adipocytes, osteoblasts, and chondrocytes revealed that 21 days post-differentiation, hpMSCs expanded in SCM and SFM were able to generate lipid vacuole deposits in adipogenesis differentiation condition, to originate bone-like nodules with calcium deposits in osteogenic differentiation condition, and to form chondroitin in chondrogenic differentiation condition (Fig. 1B). Analysis of the immunophenotypic profile of hpMSCs expanded in SFM and SCM by flow cytometry revealed that the cells exhibited conventional MSC surface markers [32]. Specifically, a positive expression of CD44, CD73, CD90, and CD105 was observed in more than 90% of cells which lacked the expression (< 5% positive) of CD14, CD19, CD34, CD45, and HLA-DR (Fig. 1C). These data suggest that the isolated and expanded hpMSCs had MSCs traits (Fig. 1D).

### Characterization of hpMSC-derived exosomes

**Fig. 2:**
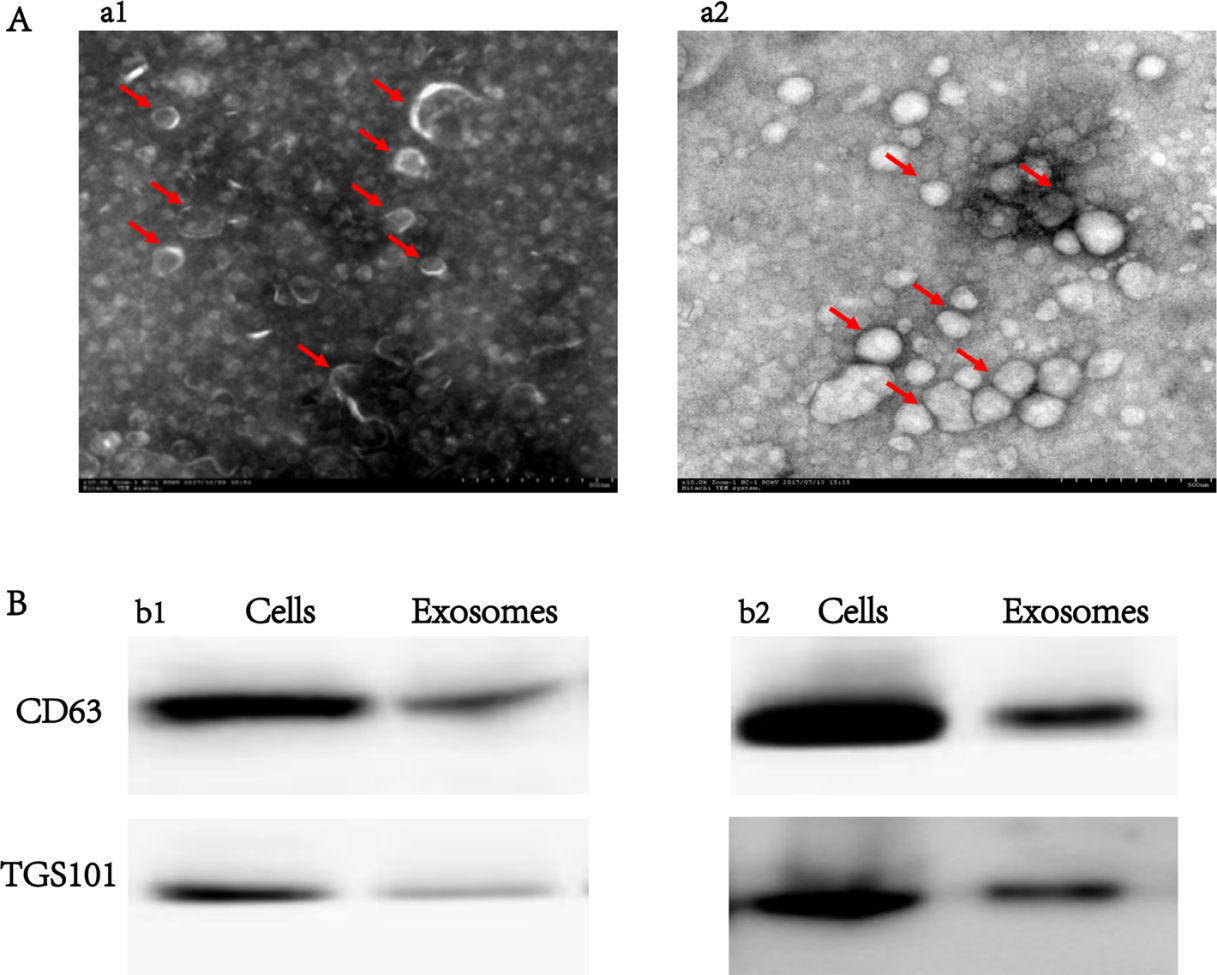
hpMSC-derived exosomes were successfully isolated by ultracentrifugation. **A** TEM analysis of hpMSC-derived exosomes obtained by (a1) ultracentrifugation and (a2) PEG precipitation (scale bars 500 nm). **B** Western blot analysis of membrane protein markers of exosomes obtained by (b1) ultracentrifugation and (b2) PEG precipitation

Assessment of the morphologies of hpMSC-derived exosomes revealed that the exosomes obtained by both techniques had a spherical shape with a diameter range of 100–200 nm and an obvious bilayer membrane structure (Fig. 2A-a1). However, the surface of the exosomes obtained using the PEG-based approach was coated with a silk-like PEG film (Fig. 2A-a2). Western blot analysis revealed that the proteins extracted from the exosomes using both techniques expressed the typical exosome markers CD63 and TGS101 (Fig. 2B). These results showed that the ultracentrifugation-derived exosomes were more suitable than those purified using the PEG-based approach and therefore were used in the subsequent experiments.

### hpMSC-derived exosomes enabled the regeneration and functional recovery of spinal cord injured animals

**Fig. 3:**
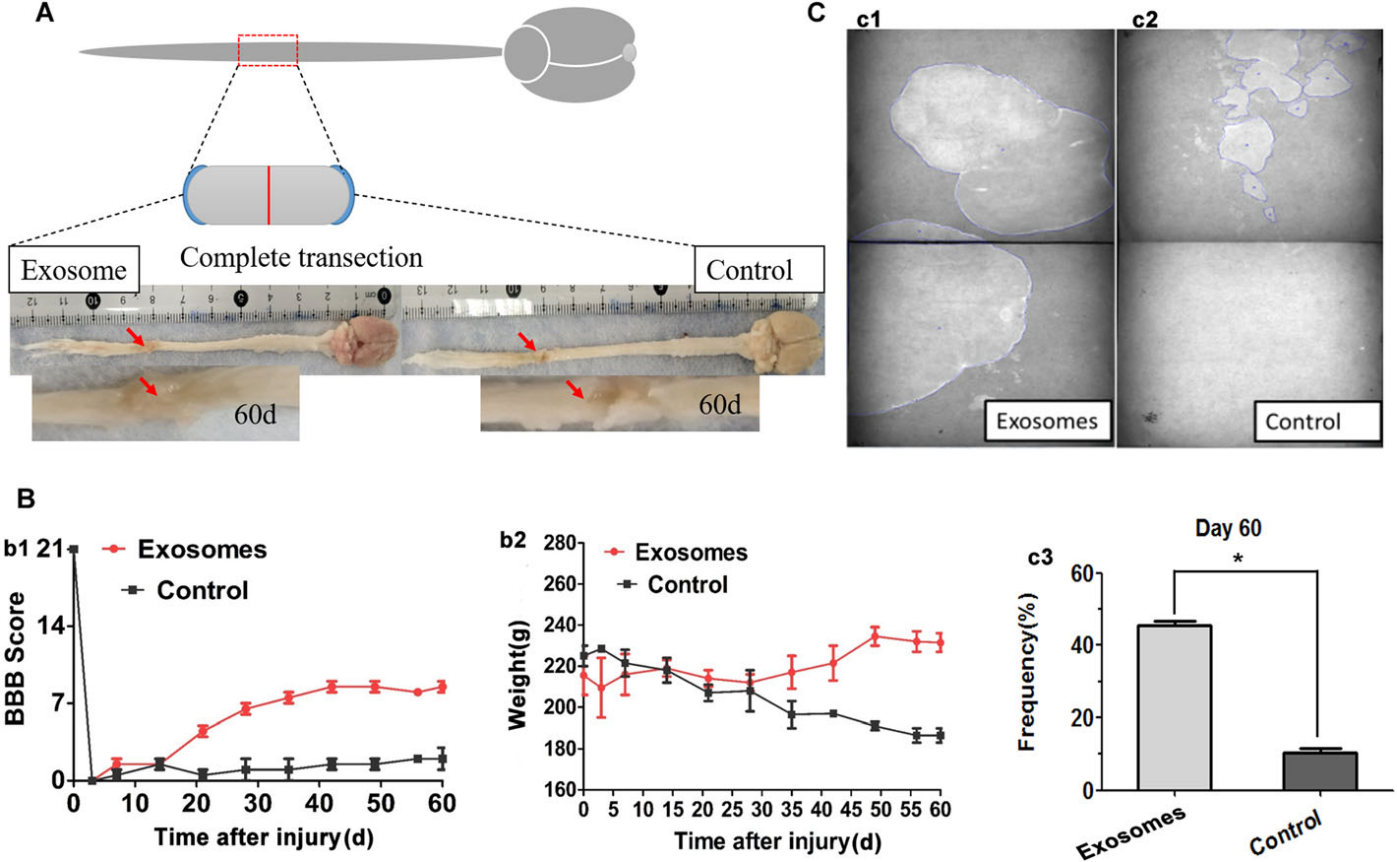
hpMSC-derived exosomes facilitated the regeneration and functional recovery after complete SCI. **A** Representative images of the spinal cord dorsal view at day 60 post complete spinal cord transection of SCI-exosomes experimental group and SCI-control group. **B** Behavioral assessment of the locomotor function progression (b1) and weight fluctuation (b2) of the animals from SCI-exosomes experimental and SCI-control groups throughout the experimental period. **C** Representative spontaneous voiding patterns of animals measured on the 60th day post-surgery of SCI- exosomes experimental group (c1) and SCI-control group (c2) and quantification of urine spot areas (c3). *p < 0.05 was considered significantly different (*n* = 6 for both groups)

Assessment of the effect of hpMSC-derived exosome transplantation revealed that SCI-exosome animals exhibited enhanced anatomic and functional recovery. Gross anatomic evaluation of the spinal cord injury revealed that the spinal cord ends of SCI-exosomes animals fused to form a smooth nerve bundle structure. Contrarily, in the SCI-control group, the lesion area was occupied by fibrous and cystic tissues that joined the two ends of the severed cord (Fig. 3A). Behavioral assessment of the hindlimb locomotor activity using the Basso–Beattie–Bresnahan (BBB) scores revealed that the animals from both experimental groups scored zero on the first day after the injury and around 1–2 in the following 3 days. Over the subsequent 60 days, SCI-control animals BBB scores were not higher than 2 whereas SCI-exosomes animals scores started steadily increasing from the fourteenth day after the surgery (Fig. 3B-b1). Evaluation of the animals’ weight fluctuation throughout the experimental period revealed that all injured-rats lost weight within the first 7 days post-injury. However, the animals from SCI-exosome group gradually recovered the weight loss whereas SCI-control animals body mass continued decreasing (Fig. 3B-b2).

### hpMSC-derived exosomes alleviate neurogenic bladder dysfunction after SCI

The neurogenic bladder dysfunction is an important clinical symptom after SCI [48] and is closely related to the loss of neurons in the injured spinal cord [49, 50]. Furthermore, exosomes have been demonstrated to have a neuroprotective effect and to promote axonal regeneration [51, 52]. To evaluate whether exosomes could alleviate the bladder dysfunction, the animals’ urologic functions were analyzed at the last day of the treatment using the spontaneous voiding test. The results revealed that the voiding behaviors of the two groups were very different. In the SCI-control group, the area of the urinary plaque was relatively small, and the urine spots were randomly scattered throughout the filter paper due to the loss of coordinated voiding. Contrarily, SCI-exosomes treated rats exhibited distinct urination patterns representative of free and active urination forming large and concentrated urine spots (Fig. 3C), implying that exosomes treatment contributed to the improvement of the voiding function of these animals thereby denoting its therapeutic potential in reducing urinary retention by recovering neurogenic bladder dysfunction.

### hpMSC-derived exosomes promote functional recovery by activating endogenous progenitors

**Fig. 4:**
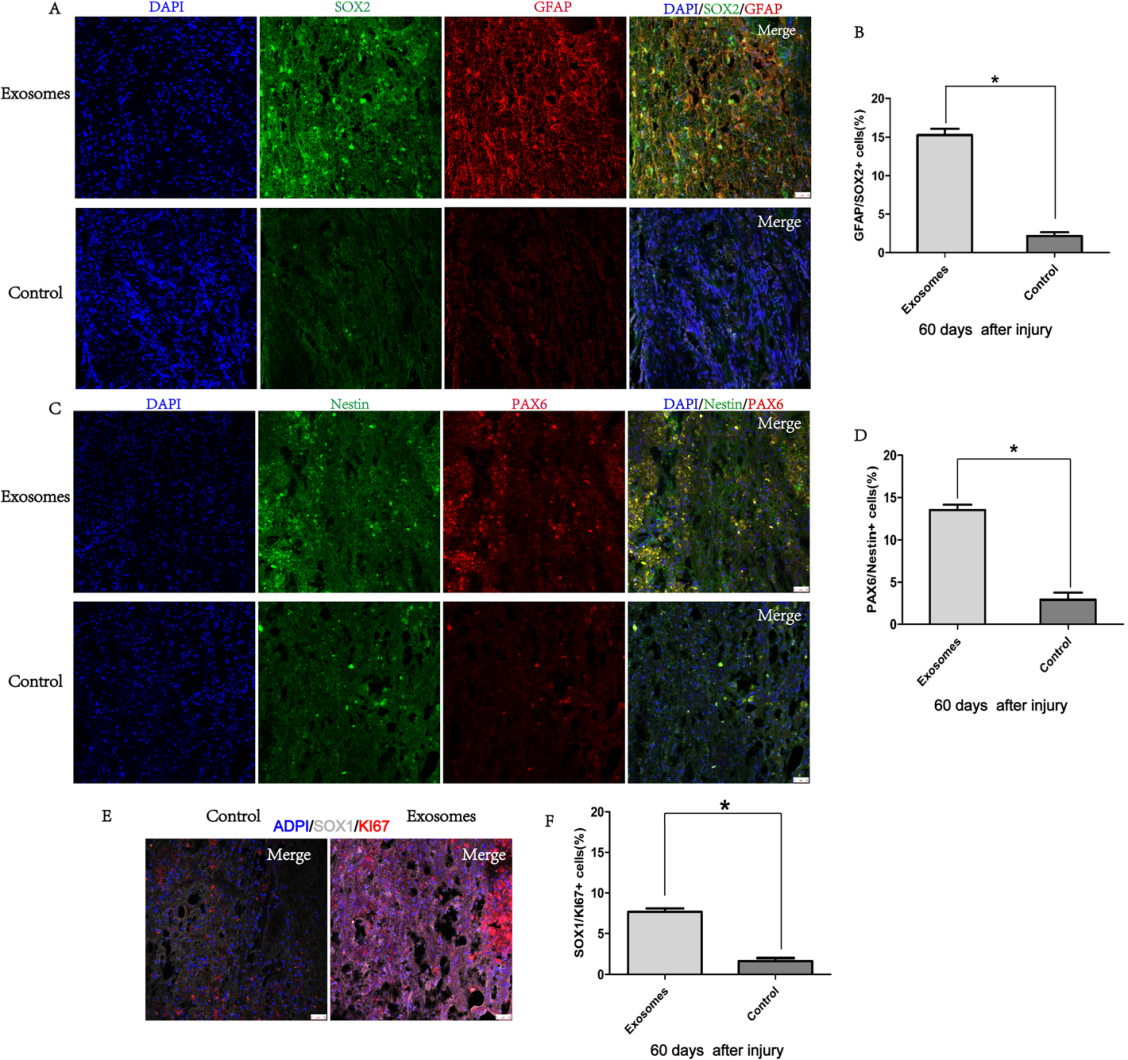
hpMSC-derived exosomes promoted the functional recovery of spinal cord injured animals by activating endogenous progenitors. **a** Representative images of GFAP and SOX2 immunofluorescence analysis. **b** Quantification of GFAP^+^SOX2^+^ cells. **c** Representative images of PAX6 and Nestin immunofluorescence analysis (scale bars 50 μm). **d** Quantification of PAX6^+^Nestin^+^ cells. **e** Representative images of SOX1 and Ki67 expression in the injured spinal cord of SCI-exosomes group and **f** SCI-control group. **g** Quantification of SOX1^+^Ki67^+^ cells. Data is presented as mean ± SD (*n* = 6). *p < 0.05 was considered significatively different

**Fig. 5:**
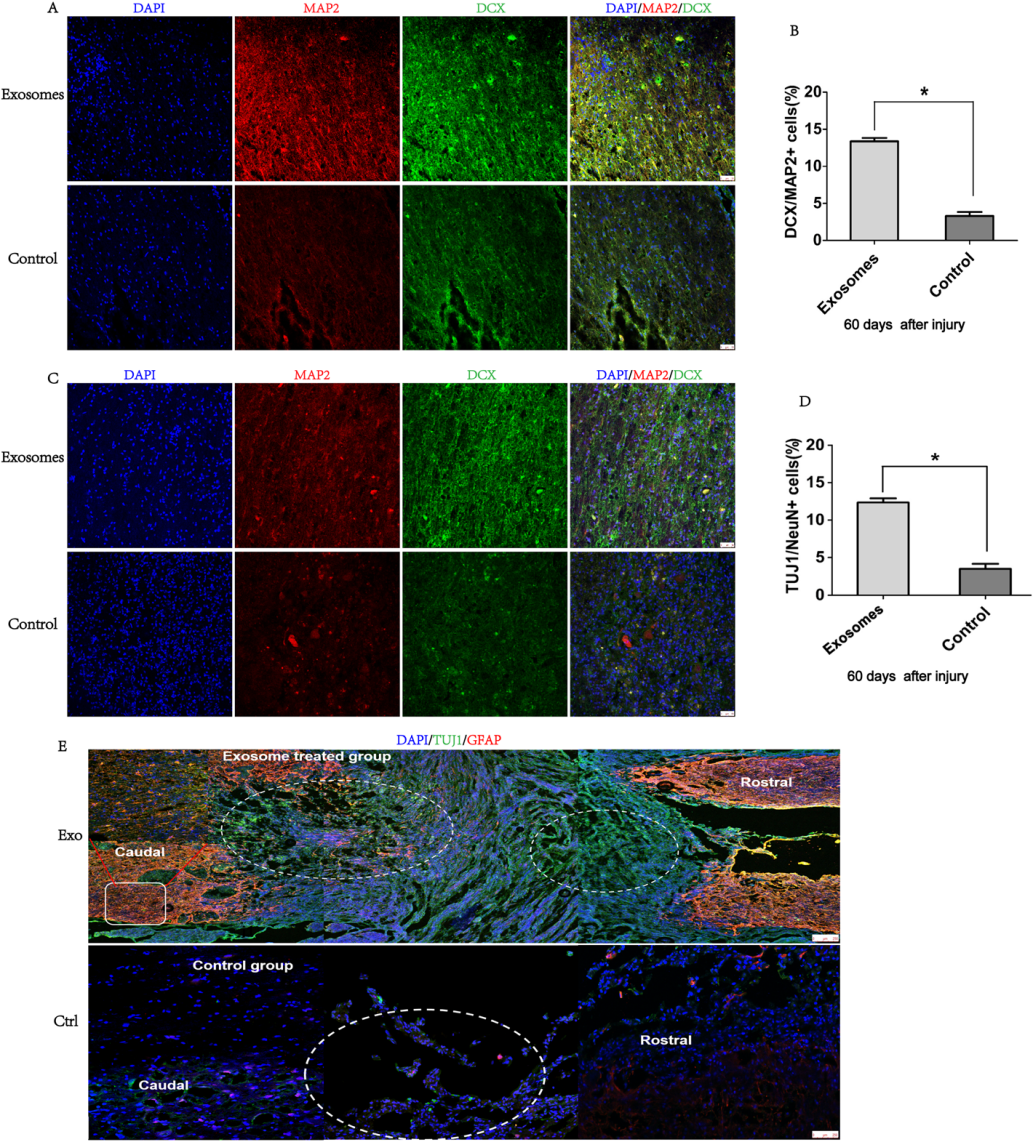
Exosomes promoted neurogenesis in the injured spinal cords. **a** Representative images of DCX and MAP 2 staining in the SCI-exosomes and SCI-control groups. **b** Quantification of DCX^+^MAP 2^+^ cells in the injured spinal cord. **c** Immunofluorescence staining of mature neuron cell markers, TUJ1 and NeuN, in the injured spinal cords of SCI-exosomes and SCI-control groups. **d** Quantification of TUN1^+^NeuN^+^ cells in the injured spinal cords (scale bars 50 μm). **e**, **f** Representative images of Tuj1 and GFAP staining in the SCI-exosomes and SCI- control groups (scale bars 250 μm). Data is presented as mean ± SD (*n* = 6). *p < 0.05 was considered was significantly different

To study the mechanisms underlying hpMSC-derived exosome therapeutic action we proceeded to the evaluation of the expression of specific markers for neural stem/progenitor cells including SOX2, PAX6, Nestin, and GFAP in the injured spinal segments. Interestingly, many SOX2 and GFAP-positive cells were detected at day 60 in the spinal cords of SCI-exosomes but not in the SCI-control group (Fig. 4a). Quantification of the data showed that the number of SOX2^+^GFAP^+^ cells was significantly higher in the SCI-exosomes group (15.8% ± 0.5% in the SCI-exosome group versus 2.8% ± 0.5% in the SCI-control group) (Fig. 4b). Accordingly, double staining of PAX6 and Nestin revealed a significantly higher percentage of PAX6^+^Nestin^+^ cells in the spinal cords of SCI-exosomes group (14.6% ± 0.5% in the SCI-exosomes group versus 3.5% ± 0.5% in the SCI-control group) (Fig. 4c, d). These results indicate that exosomes transplantation promoted the activation of endogenous neural stem/progenitor cells. Assessment of the proliferation ability of these activated NSCs by analyzing Sox1 and Ki67 expression revealed that 8% ± 0.5% of SOX1^+^KI67^+^ cells were observed at day 60 in the injured spinal cord of SCI-exosomes treated animals (Fig. 4e), while only 1.5% ± 0.5% of SOX1^+^KI67^+^ cells were detected in the SCI-control group (Fig. 4f). Statistical analysis confirmed the significant differences between the two experimental groups (Fig. 4g). To verify if the observed functional recovery of SCI-exosomes group resulted from the neurogenesis in the injured spinal cords, the expression of DCX and MAP 2 was assessed. As expected, SCI-exosomes animals exhibited a significative higher neurogenesis (Fig. 5a) depicted by the higher percentage of DCX^+^MAP 2^+^ neurons (13.6% ± 0.5% versus 3.4% ± 0.5% of DCX^+^MAP 2^+^ in the SCI-control group) (Fig. 5b). Further verification of the ability of these newborn neurons to originate mature neurons, comprehended the expression analyses of NeuN and neuron-specific β-tubulin (Tuj1) markers. As expected, 12% ± 0.5% of TUJ1^+^NeuN^+^ matured neurons were found in the spinal cords of SCI-exosome group (Fig. 5c). In contrast, only 3.0% ± 0.5% of the cells co-expressed NeuN and TUJ1 in the SCI-control group (Fig. 5d). Furthermore, GFAP and neuron-specific β-tubulin (Tuj1) double-positive cells were occasionally observed in the regenerating sites of the group treated with exosomes (Fig. 5e), but not in the control group (Fig. 5f), suggesting the differentiation of progenitor cells into new neurons. These results provide evidences that hpMSC-derived exosomes’ ability to promote the animals’ functional recovery may occur, at least partially, through the generation of new neurons in the injured spinal cords.

### hpMSC-derived exosomes promoted the proliferation of NSCs by activating MEK/ERK/CREB signaling pathway in vitro

**Fig. 6:**
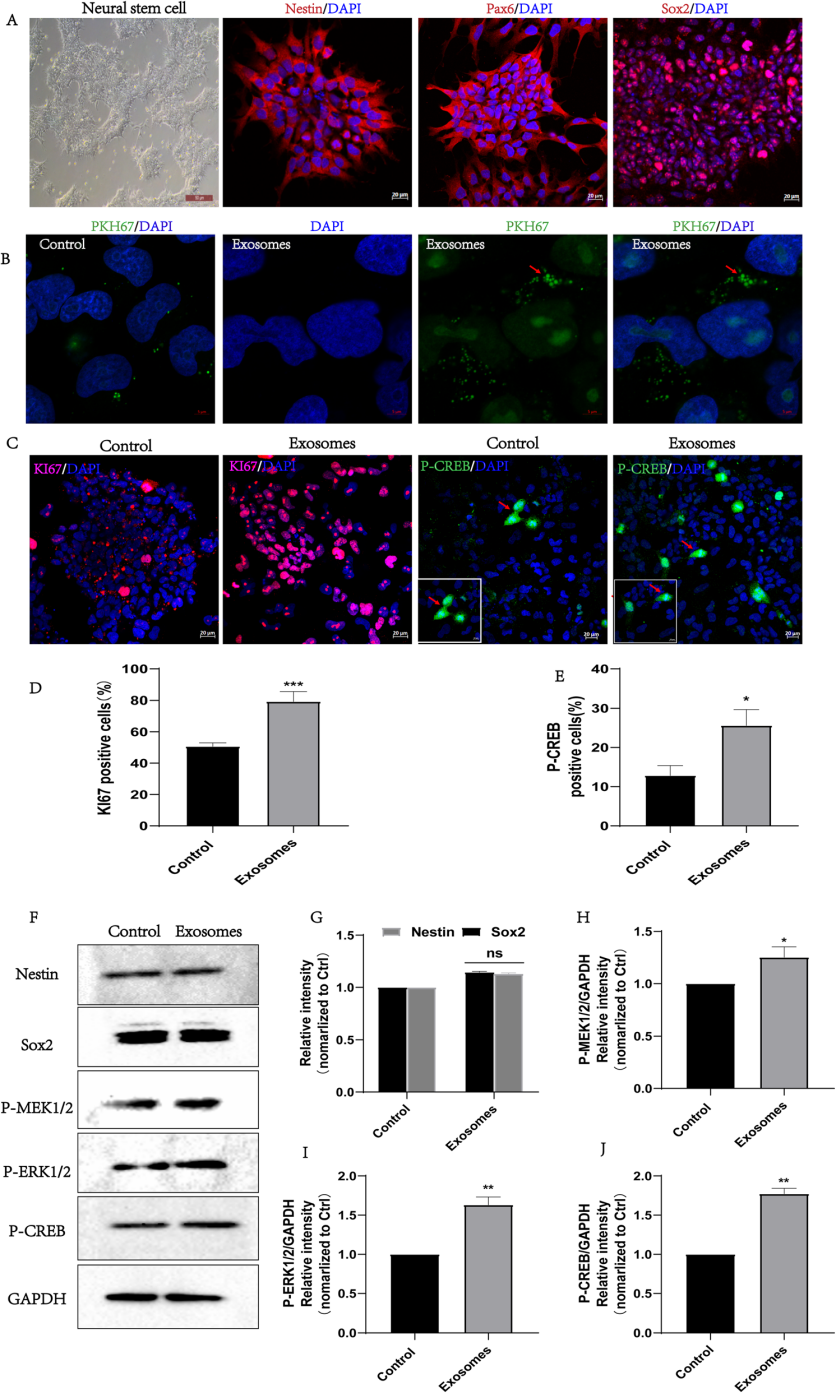
hpMSC-derived exosomes promoted the proliferation of NSCs by activating MEK/ERK/CREB signaling pathway in vitro. **a** Representative images of the rosette structures of NSCs and of the NSCs markers Nestin, Pax6 and Sox2 immunofluorescence staining (scale bars 20 μm). **b** PKH67 labeled-exosomes uptake by the cells was confirmed by confocal microscopy (scale bars 5 μm). **c** Representative images of immunofluorescence staining of Ki67 and P-CREB (scale bars 20 μm). **d**, **e** Quantification of Ki67^+^ and P-CREB^+^ cells. **f**–**j** Western blot detection of the expression of Nestin and Sox2 special markers of NSCs, and of the phosphorylation levels of MEK, ERK, and CREB. GAPDH was used as loading control. Each assay was performed in triplicate (mean ± SD, unpaired *t* test, *p < 0.05 was considered significatively different)

Several studies have reported that ERK/CREB pathway is involved in the regulation of neurogenesis [53–55]. Therefore, we hypothesized that the proneurogenic effect of hpMSC-derived exosomes could occur via the activation of this signaling pathway. To determine whether this pathway is regulated by hpMSC-derived exosomes, we proceeded to the culture of NSCs which were subsequently incubated with the isolated exosomes at a concentration of 10 μg/ml for 24 h. NSCs neuronal phenotype was confirmed by the formation of rosette structures and the positive expression of the NSCs markers Pax6, Sox2, and Nestin (Fig. 6a, f, g). Subsequent incubation of the NSCs with PKH26-labeled exosomes showed their successful uptake by the cells (Fig. 6b) which promoted the proliferation of NSCs as indicated by the increased number of positive Ki67 cells in comparison with the control group (Fig. 6c, d). Furthermore, the number of P-CREB-positive cells was significantly higher in the NSCs treated with exosomes (Fig. 6c–e). Further analysis by western blot indicated that exosomes treatment promoted the phosphorylation levels of MEK1/2, ERK1/2, and CREB (Fig. 6f, h, i, and j), indicating that their beneficial effect on NSCs proliferation might be mediated, at least in part, via activation of MEK/ERK/CREB signaling pathway which is also possibly involved in the SCI recovery observed in vivo.

## Discussion

This study provides evidence of the potential of hpMSC-derived exosomes to promote the regeneration and functional repair in CNS injuries. We were able to successfully establish a protocol to purify exosomes secreted from hpMSCs that promoted the activation of endogenous NPCs, further inducing neuronal differentiation and improving the hindlimb locomotor function and bladder dysfunction in an experimental animal model of SCI. Further exploration of the putative mechanisms, showed that in vitro, the exosomes were internalized into the NSCs promoting their proliferation via activation of MEK/ERK/CREB pathway signaling.

In recent years, the use of MSC-exosomes in the treatment of spinal cord injured patients has been gathering great attention. Containing a variety of proteins, lipids, and nucleic acids, the therapeutic advantage of using MSC-exosomes in the repair of SCI has been reported to occur via promotion of angiogenesis and axonal growth, regulation of inflammation, immunossupression, inhibition of apoptosis, and maintenance of the blood-spinal cord barrier integrity [56]. Exosomes can be extracted from different tissues such as bone marrow, adipose tissue, umbilical cord, and the placenta amniotic membrane, with the majority of the SCI repair studies reporting the use of bone marrow as the main source [17]. The use of placenta comparatively to other adult sources has the advantage that being a transient organ that is usually regarded as medical waste, it allows the extraction of a relatively large amount of cells without requiring the use of invasive methods or rising ethical concerns [56]. Moreover, the proliferative potential of MSCs derived from the placenta has been reported to be superior to the ones derived from the bone marrow [9].

To the best of our knowledge, this is the first study reporting the ability of hpMSC-derived exosomes to promote the activation of endogenous NPCs and neurogenesis and promote the functional recovery of the motor and autonomic functions after complete SCI. Intravenous injection of hpMSC-derived exosomes significantly increased the spinal expression of the neural stem/progenitor cell markers SOX2^+^GFAP^+^ and PAX6^+^Nestin^+^ cells, which was accompanied by a significant increase of NPCs proliferation ability, denoted by the increased expression of SOX1^+^KI67^+^ cells. Importantly, the functional recovery of the animals treated with the exosomes was associated to a significantly higher neurogenesis in the injured spinal cord depicted by the higher percentage of DCX^+^MAP 2^+^ neurons. Further analysis revealed a higher ability of these newborn neurons to give rise to mature neurons. The proneurogenic effect of MSC-exosomes was only previously reported in a study in which bone marrow MSC-derived exosomes were used as delivery carriers to transport miR-126 in a contusion SCI animal model. In this study, the authors showed that exosomes loaded with miR-126 promoted angiogenesis and neurogenesis, attenuated apoptosis, and promoted functional recovery [57]. The development of a novel approach able to promote the generation of new neurons through the activation of endogenous NPCs thereby stimulating the functional recovery after SCI poses as a promising and much needed therapeutic strategy. Endogenous neurogenesis has been reported to have a significant role after SCI with studies showing that the activation of endogenous NPCs may contribute to the functional recovery of the damaged nerves [58]. In another study, the use of hpMSC-derived exosomes in SCI repair was shown to enhance angiogenesis and improve the neurologic functions of mice with a contusive lesion [59]. In spite of the use of different spinal cord lesion models, it is possible that the effect of the hpMSC-derived exosomes in the activation of endogenous NPCs and neurogenesis was accompanied by an enhancement of angiogenesis that should be assessed in future studies.

In vitro, the exosomes were internalized into NSCs and promoted their proliferation via activation of MEK/ERK/CREB pathway signaling. Exosomes increased the phosphorylation of MEK, ERK, and CREB, indicating its stimulatory effect on the activity of these enzymes. In a recent study, exosomes secreted by somatic cells-induced NPCs were also shown to be able to promote the proliferation of NPCs by activating MEK-ERK signaling pathway [60]. Moreover, MEK/ERK signaling was reported to promote the proliferation of adult spinal cord NPCs and its activation was shown to be necessary for the occurrence NPCs differentiation towards the neuronal lineage [61, 62].

**Fig. 7:**
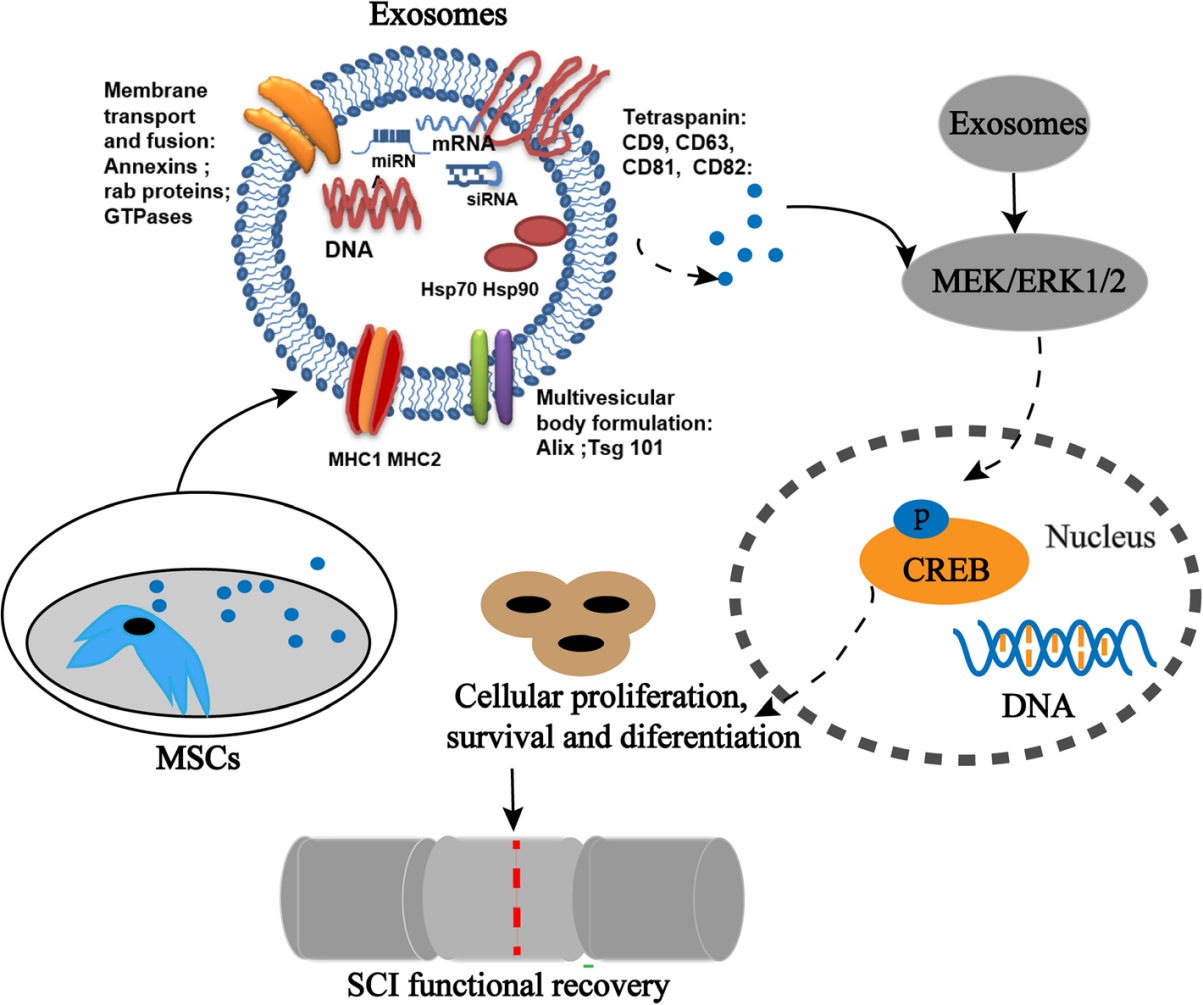
The therapeutic potential of hpMSC-derived exosomes in SCI recovery and underlying mechanisms (modified from reference [36]). hpMSC-derived exosomes were obtained from hpMSCs through ultracentrifugation. Exosomes stimulated the proliferation of NPCs via activation of MEK/ERK/CREB phosphorylation in vitro which is likely to be mediating their promoting effect on the proliferation of endogenous NPCs and differentiation into matured neurons leading to the functional recovery of SCI observed in vivo

Taken together, this study provides evidence that hpMSC-derived exosomes’ ability to promote the recovery of motor and autonomic functions after complete SCI is likely to occur through endogenous NPCs activation and neurogenesis, a process that may involve the activation of MEK/ERK/CREB signaling (Fig. 7).

## Conclusion

Our findings demonstrate that hpMSC-derived exosomes have the potential to become important therapeutic tools for the treatment of CNS injury disease as they could be used to modulate endogenous NPCs and enhance neurogenesis to promote the functional recovery after SCI.

## Data Availability

Not applicable.

## Abbreviations

BSCB: Blood-spinal cord barrier
CNS: Central nervous system
CREB: cAMP response element binding
DMEM: Dulbecco’s modified Eagle’s medium
ERK: Extracellular signal-regulated kinases
hp-MSCs: Human placental mesenchymal stem cells
MEK: Mitogen-activated protein kinase kinase
MSCs: Mesenchymal stem cells
NPCs: Neural progenitor cells
NSCs: Neural stem cells
PBS: Phosphate buffered saline
PEG: Polyethylene glycol
PFA: Paraformaldehyde
SCI: Spinal cord injury
SCM: Serum-containing medium
SFM: Serum-free medium
TEM: Transmission electron microscopy
